# Exploring the Effect of an 8-Week AI-Composed Exercise Program on Pain Intensity and Well-Being in Patients With Spinal Pain: Retrospective Cohort Analysis

**DOI:** 10.2196/57826

**Published:** 2025-02-18

**Authors:** Annika Griefahn, Florian Avermann, Christoff Zalpour, Robert Percy Marshall, Inés Cordon Morillas, Kerstin Luedtke

**Affiliations:** 1 Department of Physiotherapy, Institute of Health Sciences, University of Lübeck, Lübeck, Germany; 2 Faculty of Business Management and Social Sciences, Hochschule Osnabrück, Albrechtstraße 30, Osnabrück, Germany, 49 541969 ext 2998; 3 Evidence and Evaluation Department, medicalmotion GmbH, München, Germany; 4 Medical Department, RasenBallsport Leipzig GmbH, Leipzig, Germany; 5 Department of Orthopedic and Trauma Surgery, Martin Luther University Halle-Wittenberg, Halle, Germany

**Keywords:** exercise, mHealth, app engagement, spinal pain, artificial intelligence, AI, intensity, well-being, mobile health, apps, applications, retrospective analysis, physical activity, adults, questionnaire

## Abstract

**Background:**

Spinal pain, one of the most common musculoskeletal disorders (MSDs), significantly impacts the quality of life due to chronic pain and disability. Physical activity has shown promise in managing spinal pain, although optimizing adherence to exercise remains a challenge. The digital development of artificial intelligence (AI)-driven applications offers a possibility for guiding and supporting patients with MSDs in their daily lives.

**Objective:**

The trial aimed to investigate the effect of an 8-week AI-composed exercise program on pain intensity and well-being in patients with spinal pain. It also examined the relationship between exercise frequency, pain intensity, and well-being. In addition, app usage frequency was examined as a proxy for app engagement.

**Methods:**

Data from users who met the inclusion criteria were collected retrospectively from the medicalmotion app between January 1, 2020, and June 30, 2023. The intervention involved the use of the medicalmotion app, which provides 3‐5 personalized exercises for each session based on individual user data. The primary outcomes assessed pain intensity and well-being using the numeric rating scale (NRS) and the Likert scale. Data were collected at baseline (t0), 4 weeks (t1), and 8 weeks (t2). The correlation between exercise frequency, pain intensity, and well-being was analyzed as a secondary outcome. In addition, average session length and frequency were measured to determine app engagement. Statistical analysis included ANOVA and Spearman correlation analysis.

**Results:**

The study included 379 participants with a mean age of 50.96 (SD 12.22) years. At t2, there was a significant reduction of 1.78 points on the NRS (*P*<.001). The score on the Likert scale for well-being improved by 3.11 points after 8 weeks. Pain intensity showed a negative correlation with the number of daily exercises performed at t1 and t2. Well-being had a small negative correlation with the average number of exercises performed per day. The average number of exercises performed per day was 3.58. The average session length was approximately 10 minutes, and the average interaction with the app was 49.2% (n=27.6 days) of the 56 available days.

**Conclusions:**

Overall, the study demonstrates that an app-based intervention program can substantially reduce pain intensity and increase well-being in patients with spinal pain. This retrospective study showed that an app that digitizes multidisciplinary rehabilitation for the self-management of spinal pain significantly reduced user-reported pain intensity in a preselected population of app users.

## Introduction

Musculoskeletal disorders (MSDs) can affect muscles, tendons, cartilage, ligaments, and nerves [1]. MSDs are a major cause of chronic pain, physical disability, and loss of quality of life worldwide. They include more than 150 conditions and syndromes [2]. For example, one of the most common MSDs is spinal pain, and more specifically, low back pain (LBP) [2]. The lifetime prevalence of LBP is high, ranging from 75% to 85% [3]. Nonspecific LBP, characterized by the absence of a clear anatomical cause, accounts for approximately 90% of LBP cases [3]. Numerous risk factors for LBP have been identified, including age, obesity, sedentary lifestyle, poor posture, smoking, and psychosocial factors such as stress and depression [4]. These factors contribute to LBP’s complex and multifactorial nature, including physical, psychological, occupational, and lifestyle influences [4].

Several studies have shown that regular physical activity is effective in reducing chronic spinal pain and is consistently recommended in clinical guidelines for the management of nonspecific LBP [5-8]. A systematic review by Jordan et al [9] assessed the effects of interventions that may improve adherence to exercise and physical activity. A total of 42 trials were included, mainly focusing on patients with knee osteoarthritis and spinal pain with relatively short follow-up measurements. Promising strategies to improve exercise adherence included supervised exercise, individualized exercise, refresher or follow-up sessions, the provision of supplementary materials such as audio- or videotapes with graded exercises, self-management programs, and cognitive behavioral techniques [9].

The Clinical Practice Guideline on Intervention for the Management of Acute and Chronic LBP suggests that specific complaints and causes should be considered when selecting exercises [10]. For example, for acute complaints involving leg pain, specific exercises to activate the trunk muscles and improve the strength as well as the endurance of the back muscles should be considered. For chronic spinal pain, exercises designed to improve movement control should be included [10].

The increasing digitalization of health care and therapeutic services allows patients to use various mobile and web applications to manage their conditions [11]. Digital solutions provide a scalable and widely accessible approach, enabling the management of spinal pain in rural areas where the availability of physiotherapists is limited [12]. Digital therapeutic care applications mostly include video-based treatment programs and educational materials [13]. Not surprisingly, the number of studies investigating digital interventions has increased in recent years, including studies on the effectiveness of digital exercises in patients with spinal pain [14-18].

Another aspect of digitalization is integrating artificial intelligence (AI) methods into digital therapy applications. AI can be used to better address the heterogeneity of patients with MSDs and to personalize therapy. The use of AI in health care, particularly to support diagnosis, has increased in recent years [19]. Few studies have investigated the effects of AI-based exercises [20-22]. In addition, maintaining an exercise routine is an important factor in reducing the risk of recurrence [7,23,24]. Nearly 70% of people who recover from spinal pain experience another episode within 12 months [25]. Most maintenance strategies involve supervised group exercise, with or without equipment. However, this can be costly for the individual and the health care system [7]. Therefore, an approach using digital health applications and AI offers a new perspective to increase individual adherence to exercise at a lower cost, which could reduce the risk of recurrent pain episodes in the future [26].

To increase knowledge and provide additional evidence on the use of digital applications, the following four research questions were defined: (1) What are the effects of an 8-week app-based AI-composed exercise program in patients with spinal pain regarding pain intensity? (2) What are the effects of an 8-week app-based AI-composed exercise program on patients with spinal pain regarding their well-being? (3) Is there a correlation between the frequency of exercise and pain intensity and well-being? (4) What are the parameters of app engagement, in terms of average session duration and frequency, for patients with spinal pain in an 8-week app-based AI-composed exercise program?

## Methods

### Ethical Considerations

The study was conducted in accordance with the ethical principles of the Declaration of Helsinki. All procedures and materials used in this study were approved by the ethics committee of the University of Applied Sciences Osnabrueck (HSOS/2022/1/2). The data provided by medicalmotion GmbH were anonymized and stored on a password-protected server at the University of Applied Sciences Osnabrueck. All users consented to the collection of data by agreeing to the terms and conditions of the use of medicalmotion.

### Trial Design

The study is reported following the STROBE (Strengthening the Reporting of Observational Studies in Epidemiology) guideline (Multimedia Appendix 1) [27]. The study was registered a priori via the Open Science Framework [28]. The study is a retrospective analysis of user data. Users were recruited to the medicalmotion app between January 1, 2020, and June 30, 2023, through various recruitment opportunities, such as health insurance companies or physiotherapists.

### Participants

Inclusion criteria were as follows: (1) men and women who reported spinal pain (including upper and lower back) in the initial therapeutic questionnaire, (2) individuals aged between 18 and 65 years with access to the medicalmotion app, and (3) participants who had at least 8 interactions with the medicalmotion app during the 8-week evaluation period. Exclusion criteria were as follows: (1) persons diagnosed with a neurological or mental illness, (2) persons currently suffering from an infection or systemic disease, or (3) persons regularly taking medication for mental illness. The sample for this study consisted of all enrolled participants who met the inclusion criteria.

### Intervention

The medicalmotion GmbH offers a comprehensive mobile and web application for various pain conditions. The mobile app not only offers AI-based exercises but also includes a wide range of additional features, including relaxation exercises, podcasts, a chat function, and a health cockpit, which tracks the user’s condition throughout use. The AI-chosen exercises consider medical history data, including pain characteristics, lifestyle information, and well-being. In addition, user feedback from previous exercise sessions is incorporated into the composition process to ensure a personalized approach.

The system dynamically selects the most appropriate exercises based on real-time data such as the user’s pain location and intensity, comfort level, and feedback from previous exercises. It creates an exercise needs profile and matches it with exercises from an extensive database. This adaptive approach allows adjustments for acute pain or changing circumstances. The user has the option to choose the number of daily exercises (3 to 5), all of which are delivered as real-time audio-based exercise videos. Each exercise has an average length of 167 seconds. The system also identifies potential causes of pain and adapts the exercise composition accordingly, ensuring a personalized exercise program with transparent traceability. Its training repertoire consists of a selection of 300 exercises. These exercises are categorized into 3 groups: release, mobility, and strength. Release exercises aim to induce muscle and tissue relaxation. Mobility exercises involve the full spectrum of body movement with complex movement patterns. Strength exercises strengthen muscles, tendons, and supporting tissues. An example of each type of exercise is included in Multimedia Appendix 2.

### Outcomes

The primary outcome measure was pain intensity. An 11-point numeric rating scale (NRS) assessed participants’ pain intensity. A change of 1‐2 points was considered clinically relevant [29]. The secondary outcome was well-being, measured using an 11-point Likert scale. A score of zero indicates poor well-being, while a score of 10 indicates perfect well-being. Data were collected at baseline (t0), 4 weeks (t1), and 8 weeks (t2). In addition, the following parameters were collected at t0 via a therapeutic questionnaire as part of the onboarding within the medicalmotion app: age, work mode, sports frequency, and sex. The initial pain areas’ anatomical location and duration were recorded using a body map. Pain duration was categorized into chronic, subacute, and acute. The anatomical location of pain was recorded for the buttocks, lower back, upper back, and neck areas. Multiple responses were allowed for anatomical location. Data collection included the weekly training volume, the number of training sessions with more than one exercise, the number of training sessions in which all exercises were completed, and the total number of exercises completed. The application evaluated the average length and frequency of sessions to assess engagement with the app. Data were only collected within the medicalmotion app.

### Statistical Analysis

For the statistical analysis, an ANOVA with repeated measurement was conducted to examine the effects of the 8-week app usage on pain intensity and well-being. If the assumption of sphericity was not met, the Greenhouse-Geisser correction was used. For post hoc analysis, the Bonferroni correction was used.

In addition, Pearson correlation (*r*) between pain intensity, well-being, average number of exercises per day, total number of exercises, and skipped exercises was examined. If there was no linear correlation, Spearman correlation (ρ) was performed. The interpretation of the correlation coefficients was based on Cohen thresholds [30].

The average session length was calculated by multiplying the average number of exercises performed by the average exercise duration of 167 seconds. Session frequency was calculated by dividing the total number of active days by the total number of available days (56 days). Session frequency was expressed as a percentage. Session length was given in seconds and minutes.

The significance level was set at *P*=.05. The effect size was described by partial η² [30]. Results are reported as mean, SD, minimum, maximum, CIs, *F* value, and *df*. Statistical analysis of the data was performed using IBM SPSS Statistics version 29.

## Results

### Descriptive Results of the Participants

The total sample included 379 participants, of whom 138 (36.4%) were men and 241 (63.6%) were women. The mean age was 50.96 (SD 12.22) years. Data on mode of work, frequency of sport, initial pain areas, and completed exercises per day are shown in Table 1.

**Table 1.** Descriptive results of the cohort.

Variables			Values
Categorical variables			
	Participants, n (%)		
		Female	241 (63.6)
		Male	138 (36.4)
	Work mode, n (%)		
		Sitting and standing	165 (43.5)
		Sitting	177 (46.7)
		Hard work	17 (4.5)
		Standing	20 (5.3)
	Sport frequency, n (%)		
		Never	59 (15.6)
		1‐3 times per week	248 (65.4)
		>3 times per week	72 (19)
	Initial pain areas (anatomical location), n (%)		
		Lower back	229 (60.4)
		Neck	201 (53)
		Buttocks	109 (28.8)
		Upper back	184 (48.5)
Continuous Variables			
	Age (years), mean (SD)		50.96 (12.22)
	Number of initial areas of pain based on the duration of the pain, mean (SD)		
		Total	4.86 (4.02)
		Chronic pain areas (>6 months)	2.43 (3)
		Subacute pain areas (>1 week and <6 months)	1.52 (2.18)
		Acute pain areas (<1 week)	0.91 (2.33)

### Pain Intensity

**Figure 1.**
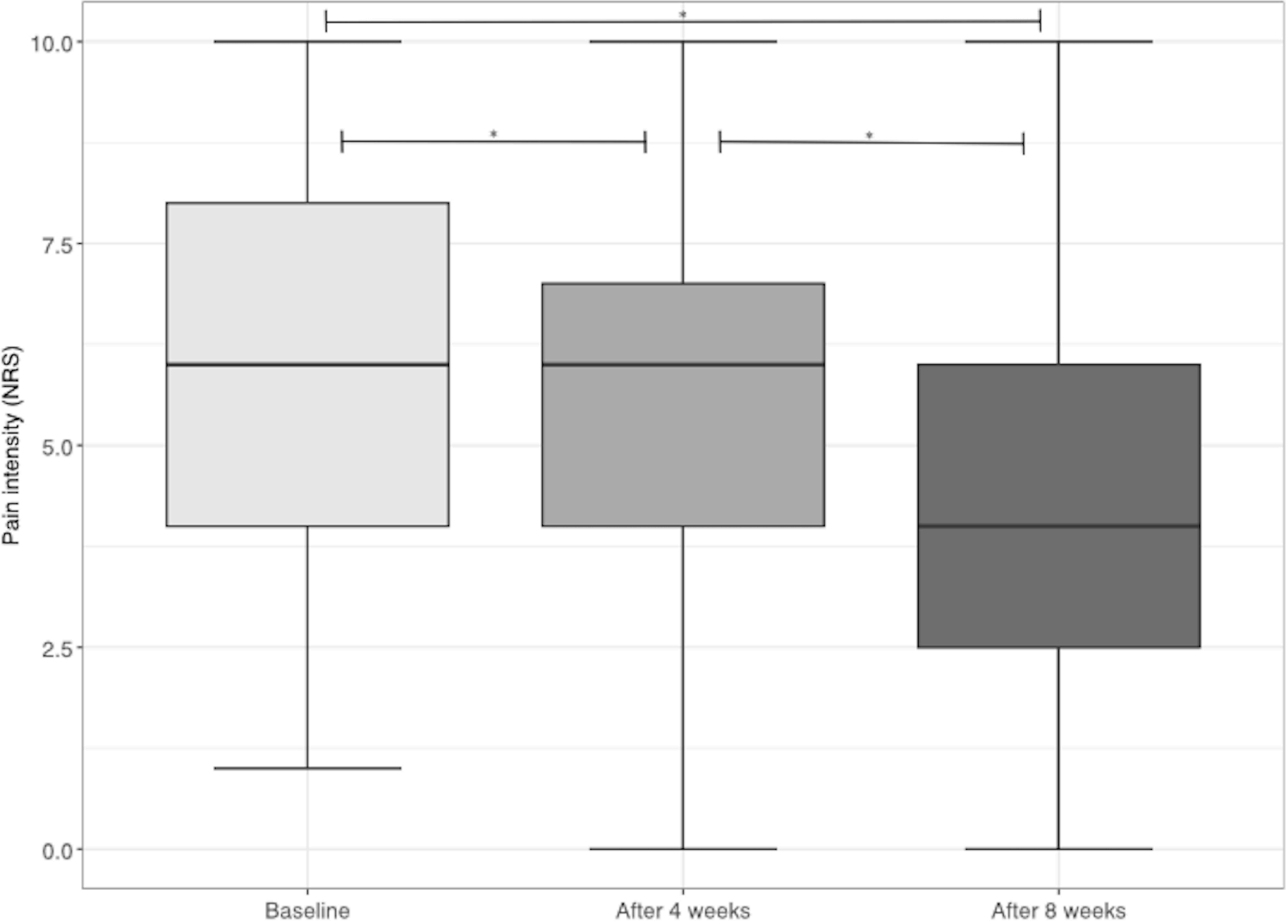
Changes in pain intensity over time. An asterisk (*) denotes a significant difference. NRS: numeric rating scale (from 0 to 10); lower whisker: minimum; upper whisker: maximum.

At t0, the intervention group showed a mean pain intensity of 6.08 (SD 2.16) on the 0‐10 NRS (Table 2). After 8 weeks (t2), the intervention group showed a mean reduction of 1.78 (SD 2.05) points on the 0‐10 NRS (Figure 1). The factor “time” was significant (*F*_1.88,710.96_=179.861, *P*<.001) and showed a large effect with η²=0.32. Post hoc analysis with Bonferroni correction showed a significant difference at each time point with *P*<.001 (Table 2).

**Table 2.** Statistical analysis of pain intensity and well-being.

Measurement points	Mean (SD)	Mean difference (SD)		Percentage difference, %		95% CI		Min	Max	*P* value	Partial η²^a^
		t0^b^	t1^c^	t0	t1	Lower	Upper				
Pain intensity (NRS^d^)										<.001	0.32
t0	6.08 (2.16)	—^e^	—	—	—	5.85	6.3	1	10	—	—
t1	5.54 (2.23)	0.53 (1.9)	—	8.3	—	5.32	5.77	0	10	—	—
t2^f^	4.3 (2.28)	1.78 (2)	1.25 (1.6)	29.3	22.4	4.07	4.52	0	10	—	—
Well-being (Likert scale)										<.001	0.64
t0	4.95 (1.65)	—	—	—	—	4.8	5.1	2	10	—	—
t1	7.07 (1.43)	2.1 (1.8)	—	42.8	—	6.92	7.22	4	10	—	—
t2	8.06 (1.38)	3.1 (1.7)	1 (1.5)	62.8	14	7.9	8.2	4	10	—	—

a η² = effect size.

b t0: baseline.

c t1: after 4 weeks.

d NRS: numeric rating scale.

e Not applicable.

f t2: after 8 weeks.

### Well-Being

**Figure 2.**
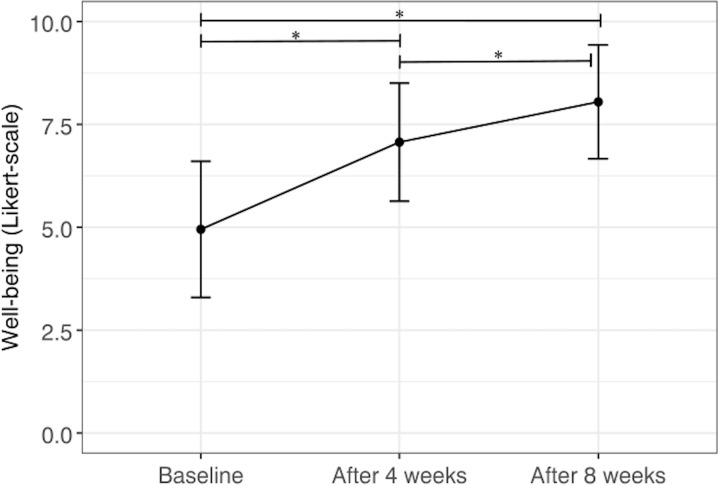
Changes in well-being over time are shown with mean and SD. An asterisk (*) denotes a significant difference.

At t0, mean well-being was 4.95 (SD 1.65) on the 11-point Likert scale (Table 2). After 8 weeks (t2), the well-being improved by 3.11 points (Figure 2). The factor “time” was significant (*F*_1.94,732.57_=671.97, *P*<.001) and showed a large effect with η²=0.64. Post hoc analysis with Bonferroni correction showed a significant difference at each time point with *P*<.001 (Table 2).

### App Engagement

The average session length was 597.86 seconds (~10 minutes). The medicalmotion app was used on an average of 49.2% (27.6 days) of the 56 available days. Table 3 shows the variables: active days, total number, and average number of exercises performed.

**Table 3.** Overview of the average activity within the medicalmotion app.

Variable	Mean (SD)	Min	Max
Active days within 8 weeks trial (days)	27.54 (10.98)	16	57
Finished exercises (number)	99.34 (49.9)	1	279
Skipped exercises (number)	1.36 (4.87)	0	67
⌀ finished exercises per day (number)	3.58 (0.9)	1	5

### Correlation Between Pain Intensity and Exercise Completion

**Figure 3.**
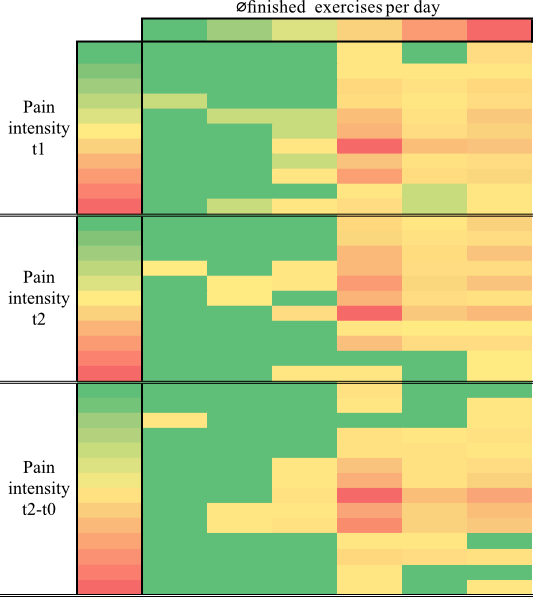
Heatmap of the Spearman correlation between pain intensity and average finished exercises per day. The x-axis represents the average number of exercises per day (0‐5), and the y-axis represents pain intensity (0‐10). The color scale from green to yellow to red represents the percentage distribution. Green means a low percentage, yellow a medium percentage, and red a high percentage.

Pain intensity levels showed a statistically significant negative correlation with the average number of exercises performed per day at t1 (*P*<.001) and t2 (*P*=.004). This suggests that as pain intensity increased, the number of exercises performed per day tended to decrease. Conversely, as pain intensity decreased, the number of exercises performed per day tended to increase. A similar, although slightly weaker, negative correlation was observed between pain intensity at t2 and the total number of exercises completed (*P*=.04). Notably, no statistically significant correlation was found between missed exercises and pain intensity. Details of the correlation coefficients and *P* values can be found in Multimedia Appendix 3. Figure 3 shows the relationship between the pain intensity at t1 and t2, the difference between t2 and t0, and the average number of exercises per day using a heat map.

### Correlation Between Well-Being and Exercise Completion

The average daily number of exercises completed showed a statistically significant, albeit small, negative correlation with the change in well-being over 8 weeks (*P*=.01). This means that as well-being increased, the number of exercises completed tended to decrease. Conversely, as well-being decreased, the average number of exercises completed tended to increase.

**Figure 4.**
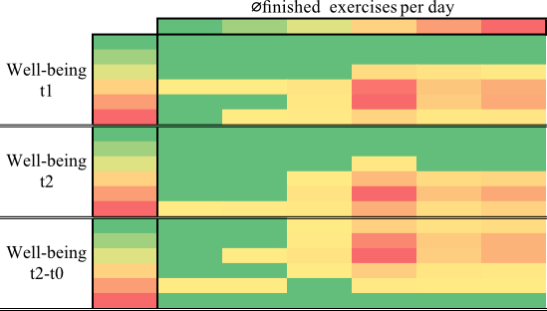
Heatmap of the Spearman correlation between well-being and finished exercises per day. The x-axis represents the average number of exercises per day (0‐5), and the y-axis represents well-being (0‐10). The color scale from green to yellow to red represents the percentage distribution. Green means a low percentage, yellow a medium percentage, and red a high percentage.

Figure 4 shows the relationship between the well-being at t1 and t2, the difference between t0 and t2, and the average number of exercises per day using a heat map.

## Discussion

### Principal Results

This study aimed to evaluate the effect of an app-based, AI-composed exercise program on pain and well-being in patients with spinal pain. A significant effect size (η²=0.32) was achieved with a clinically relevant reduction in pain intensity of 1.78 points. A similar picture emerges with regard to well-being. The observed change in well-being of 3.11 points represents a substantial improvement. In addition, the large effect size (η²=0.64) indicates that a significant proportion of the variation in well-being is attributable to the intervention. The study showed significant negative correlations between pain intensity levels at t1 (*P*<.001) and t2 (*P*=.004) and the average number of daily exercises, meaning that a higher pain intensity was associated with reduced daily exercise. A weaker negative correlation was observed between pain intensity at t2 and the total number of exercises performed (*P*=.04). The graph shows that both too much and too little exercise did not result in a significant change in pain intensity.

In addition, the average number of exercises completed per day showed a small but significant negative correlation with the change in well-being over 8 weeks (*P*=.01), suggesting that increased well-being was associated with a tendency to exercise less per day. In terms of session frequency, the medicalmotion app was used on 49.2% (27.6 days) of the 56 available days. With an average exercise duration of 167 seconds, this cohort’s average use time was approximately 10 minutes per 8-week session.

### Limitations

While this study provides valuable insights, its limitations require careful consideration. One limitation of the AI-based app is that it categorizes the exercises into strength, mobility, and release. However, it needs to be made clear whether these categories or the individualized plan contributed significantly to the observed effects. Future studies should focus on a more detailed evaluation of the specific effects of AI on outcomes.

Pain intensity (NRS) and well-being (Likert scale) were treated as metrically scaled in the statistical analysis, allowing for the calculation of repeated measures ANOVA. Therefore, the results are presented with the mean and SD. Based on the study by Nair and Diwan [31], parametric tests can be used for ordinal scaled data (pain intensity) if a normal distribution is present. This fact should be taken into account when interpreting the results.

Because of the broad inclusion criteria used, there is variability in the participants. This variability results from the wide age range and the broad categorization of spinal pain as an inclusion criterion. As a result, the population may have included individuals with a medical diagnosis of spinal pain as well as those with no diagnosis or an inconclusive diagnosis. This diversity in the study population could affect the influence of the app on the participants. In order to increase the specificity of the results and improve the assessment of effectiveness, it may be beneficial to introduce a differentiation based on diagnostic categories.

Another limitation is the data collection period, from 2020 to 2023. The COVID-19 pandemic that was ongoing during this period, with its peak years in 2020 and 2021, may have confounded the results. During this time, the amount of physical activity and opportunities for public exercise in gyms were limited. In addition, many people were experiencing psycho-emotional stress, which may have affected their perception of pain. Finally, the long-term effects of COVID-19 infection can cause heterogeneous symptom complexes, which may further influence the physical complaints of the participants.

A further limitation of this study is the short duration of the intervention, which was limited to 8 weeks. Such a short period of time limits the conclusions that can be drawn about the long-term effects of the intervention and the sustainability of the results. In particular, aspects such as long-term compliance and the associated changes in exercise frequency could not be adequately assessed during this short observation period. Future studies with longer follow-ups are needed to make reliable statements about the long-term benefits and stability of the intervention.

In the context of these limitations, it is important to acknowledge that this study has inherent drawbacks, including potential selection bias, lack of a control group, and reliance on self-reported data. These limitations may introduce bias and affect the generalizability of the findings. The retrospective cohort study design also has its own set of drawbacks, including potential data quality issues and variability in user recruitment. In particular, the lack of control for concomitant pain-relieving interventions, such as physiotherapy, adds complexity and requires caution in drawing definitive conclusions. Consequently, the results of the study should be used primarily for hypothesis generation, with validation sought through prospective study designs to address these limitations and provide a more comprehensive understanding of the issue.

### Comparison to Prior Work

These results on pain intensity reduction can be compared with home-based exercise therapy, as shown in a review by Quentin et al [32], who demonstrated a reduction in pain intensity in nonspecific LBP and a reduction in functional impairment with home-based exercise therapy and also reported a high effect size. A study by Weise et al [33] compared the effects of digital exercise therapy with physiotherapy. This study also showed a reduction in pain intensity of 2.92 points after 6 weeks in the intervention group. The results of this study showed similar effects. However, the added value of the AI-composed exercises cannot be deduced from these results. Therefore, it cannot be concluded whether the individualized exercises provided by the mobile app have added value in reducing pain or whether simply exercising induced the effect. However, a study by Cimarras-Otal et al [34] showed that exercises tailored to the needs of workers had a better outcome than general exercises.

The interaction between well-being and chronic pain has been reported previously [35]. For example, improvements in well-being have been shown to lead to reductions in pain catastrophizing and depression [35,36]. Based on the association between exercise and increased subjective well-being, the exercises provided in this study also influenced the psychological aspects of chronic pain.

Although a correlation only indicates an association and does not imply causation, the results of the correlation analysis can be interpreted in several ways. On the one hand, a reduction in pain could be the reason why users exercised more. In other words, more exercise or movement became possible with less pain. On the other hand, increased practice could also cause pain reduction; that is, users could reduce their pain through practice. Further studies are therefore needed to establish a causal relationship and the ideal frequency of exercise. The relationship between exercise frequency and changes in pain intensity was demonstrated in a large-scale study by Marshall et al [37]. Interestingly, the frequency of exercise was very similar between participants with significant improvement and those with deterioration. This fact may be due to the inclusion of different types of exercise, making it difficult to assess the effect of specific types of exercise. Therefore, an individualized training program for each person may be necessary to improve pain intensity successfully [37].

Another factor that may influence exercise frequency may be motivation. Increased pain and decreased well-being could both increase and decrease motivation to exercise. On the one hand, if you are in pain, you need to do something about it, or conversely, if you are in pain, there may be a fear or concern that exercise will only worsen the condition. Both scenarios are plausible and require further investigation in future studies. In this context, motivation to exercise should be assessed to identify possible relationships between pain intensity and motivation.

A 1985 review by Dishman et al [38] identified several factors that influence exercise frequency. Self-motivation, anticipated personal health benefits, perceived well-being, and enjoyment of physical activity positively contributed to the likelihood of participating in a supervised exercise program. Conversely, factors such as mood disturbance, health concerns, and knowledge about exercise or health negatively influenced the likelihood. This observation underscores the importance of motivation to exercise and highlights its significant influence on the frequency of engagement in physical activity.

A study by Meyer et al [39] showed a positive association between the frequency of physical activity and self-rated health. This finding contrasts somewhat with the current results, as a negative association was found between an average number of daily exercises and a change in self-rated health over 8 weeks. One possible explanation for this discrepancy could be the distinction between well-being and self-rated health. Another explanation could be that the lower the number of complaints, the lower the compliance to continue exercising. Users may have felt better and consequently exercised less because they no longer felt the need to exercise. One possible explanation may be the influence of self-motivation on training frequency, which is already a known factor for training frequency [38]. The correlation cannot answer these considerations; hence, further research is needed to establish a causal relationship [38]. In addition, the method of assessing the frequency of physical activity may also contribute to the difference. In the study by Meyer et al [39], participants provided a self-assessment of physical activity using a questionnaire, in contrast to the objective values obtained from the app in this study. Another factor to consider for the discrepancy between Meyer et al [39] and this study’s results is the study population. The aforementioned study examined healthy participants and not a pain-specific population [39].

To date, few studies have reported on the frequency of use of eHealth products. In a study by Labinsky et al [40], 9 out of 39 participants reported having only used the applications once. A study by Tian et al [41] also found that the average usage time of common digital products was between 2 and 10 minutes. No reliable values could be found specifically for eHealth or mobile health products.

### Conclusions

In summary, the study investigated the multiple effects of an 8-week, app-based, AI-composed exercise program on patients with spinal pain. It addressed critical dimensions, including pain intensity and well-being, while exploring the potential relationship between exercise frequency and these outcomes. The results highlight the importance of exercise frequency in influencing pain intensity and well-being, revealing a compelling interplay between engagement in prescribed exercises and observed improvements in health outcomes. This finding strengthens the rationale for personalized exercise programs and highlights the importance of adherence and regularity in achieving optimal outcomes.

Hypotheses that were derived from this retrospective analysis for a randomized controlled trial: (1) participants in the intervention group following an 8-week AI-composed exercise program will experience a statistically significant reduction in pain intensity and a significant improvement in well-being compared with the control group (alternative hypothesis) and (2) the frequency of engagement in the prescribed exercises will be positively correlated with decreased pain intensity and increased well-being among participants with spinal pain (correlation hypothesis).

## Supplementary material

10.2196/57826 Multimedia Appendix 1 STROBE (Strengthening the Reporting of Observational Studies in Epidemiology) statement.

10.2196/57826 Multimedia Appendix 2 Exercise examples.

10.2196/57826 Multimedia Appendix 3 Exercise-induced correlation of pain intensity and well-being.

## Abbreviations

AI: artificial intelligence
LBP: low back pain
MSD: musculoskeletal disorder
NRS: numeric rating scale
STROBE: Strengthening the Reporting of Observational Studies in Epidemiology
