# Supplementary material for: Exploring the Effect of an 8-Week AI-Composed Exercise Program on Pain Intensity and Well-Being in Patients With Spinal Pain: Retrospective Cohort Analysis
Source: JMIR Form Res. 2025 Feb 18;9:e57826. doi: 10.2196/57826 (PMC11856805; doi:10.2196/57826)
Supplement: Multimedia Appendix 2 [file formative-v9-e57826-s002.pdf]

Name: Breast Flexion
Type: Mobility

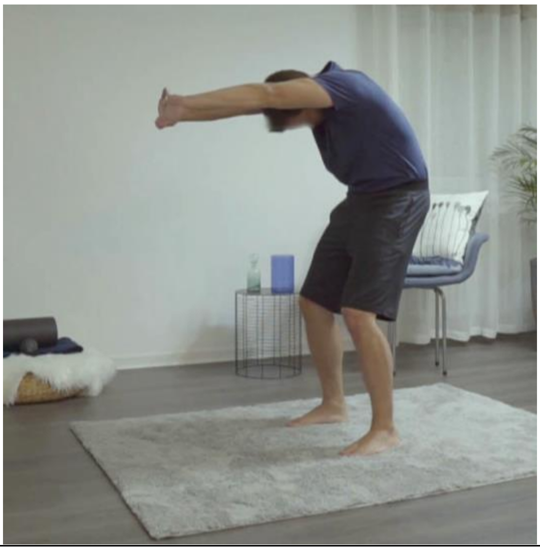

Description:
In a slightly bent posture, the thoracic spine is maximally flexed in combination with a shoulder movement.
Aim:
The targeted flexion of the entire spine is intended to promote the mobility of the spine. In addition, the targeted movement of the spine is intended to promote the mobility of the individual spinal segments.

Name: The Diagonal
Type: Release

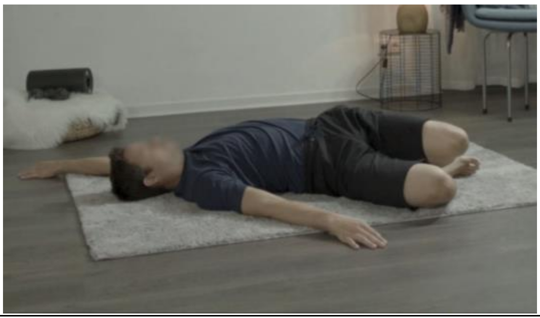

Description:
The legs are first raised and then placed sideways to the right and left. The position, with the legs lying sideways, is held for 30 seconds.
Aim:
The exercise is designed to help increase the mobility of the back and shoulder girdle by stretching the front structures in a targeted manner

Name: Active Seat
Type: Strength

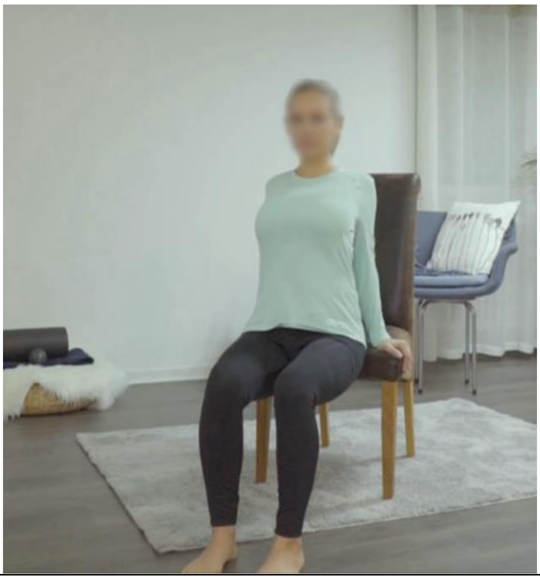

Description:
The hands are placed at the end of the seat so that the arms are supported. The sternum should be moved towards the ceiling.
Aim:
In addition to strengthening the shoulder girdle and core muscles, the aim of this exercise is to improve intermuscular coordination and mobility of the thoracic spine.
