# Supplementary material for: Exploring the Effect of an 8-Week AI-Composed Exercise Program on Pain Intensity and Well-Being in Patients With Spinal Pain: Retrospective Cohort Analysis
Source: JMIR Form Res. 2025 Feb 18;9:e57826. doi: 10.2196/57826 (PMC11856805; doi:10.2196/57826)
Supplement: Multimedia Appendix 3 [file formative-v9-e57826-s003.pdf]

Exercise-Induced Correlation of Pain Intensity and Well-being

Variable	Finished exercises	⌀ finished exercises per day	Skipped exercises	Pain intensity t1	Pain intensity t2	Pain intensity t2-t0	Well-being t1	Well-being t2	Well-being t2-t0
**Total finished exercises**									
ρ	1	0.569[a]	-0.022	-0.08	-0.104[a]	0.01	0.03	0.054	0.027
P value	-[b]	<.001	.7	.1	.04	.9	.6	.3	.6
**⌀ finished exercises per day**									
ρ	0.569[a]	1	-0.073	-0.186[a]	-0.148[a]	-0.02	-0.027	-0.035	-0.126[a]
P value	<.001	-	.2	<.001	.004	.7	.6	.5	.014
**Skipped exercises**									
ρ	-0.022	-0.073	1	0.014	0.029	0.027	-0.05	-0.064	0.089
P value	.7	.2	-	.8	.6	.6	.4	.2	.1
**Pain intensity t1**									
ρ	-0.08	-0.186[a]	0.014	1	0.7[a]	0.1[a]	0.146[a]	0.14[a]	0.122[a]
P value	1	<.001	.8	-	<.001	.04	.004	.006	.02
**Pain intensity t2**									
ρ	-0.104[a]	-0.148[a]	0.029	0.7[a]	1	0.471[a]	0.06	0.046	0.051
P value	.04	.004	.6	<.001	-	<.001	.3	.4	.3
**Pain intensity t2-t0**									
ρ	0.01	-0.02	0.027	0.1[a]	0.471[a]	1	-0.01	-0.103[a]	-0.131[a]
P value	.9	.7	.6	.04	<.001	-	.9	.045	.01
**Well-being t1**									
ρ	0.03	-0.027	-0.05	0.146[a]	0.06	-0.01	1	0.39[a]	-0.033
P value	.6	.6	.4	.004	.3	.9	-	<.001	.5
**Well-being t2**									
ρ	0.054	-0.035	-0.064	0.14[a]	0.046	-0.103[a]	0.39[a]	1	0.448[a]
P value	.3	.5	.2	.006	.4	.045	<.001	-	<.001
**Well-being t2-t0**									
ρ	0.027	-0.126[a]	0.089	0.122[a]	0.051	-0.131[a]	-0.033	.0448[a]	1
P value	.6	.014	.1	.02	.3	.01	.5	<.001	-

[a]The correlation is significant at a significance level of .05 (two-tailed), [b]Not applicable.
ρ – correlation coefficient
